# M. Bonnet

**Published:** 1859-03

**Authors:** 


OUR London exchanges also bring us the intelligence of the death of M. BONNET, a distinguished surgeon of Lyons, and author of several valuable treatises on surgical affections. He is well known by his works, "On Sections of the Tendon," "On Disease of the Articulations," "On the Practice of Cauterization," etc. M. Bonnet was about fifty years of age, and died after a short illness. The medical men of Lyons intend erecting a monument to his memory.

